# Clinical Efficacy and Safety of Low-Level Laser Therapy in Patients with Perennial Allergic Rhinitis: A Randomized, Double-Blind, Placebo-Controlled Trial

**DOI:** 10.3390/jcm10040772

**Published:** 2021-02-15

**Authors:** Hahn Jin Jung, Young-Jun Chung, Young-Seok Choi, Phil Sang Chung, Ji-Hun Mo

**Affiliations:** 1 Department of Otorhinolaryngology-Head and Neck Surgery, Chungbuk National University College of Medicine, Chungbuk National University Hospital, Cheongju 361711, Korea; hahnjin2@naver.com (H.J.J.); yschoi@chungbuk.ac.kr (Y.-S.C.); 2 Department of Otorhinolaryngology, Dankook University College of Medicine, Cheonan 31116, Korea; entdocjung@daum.net (Y.-J.C.); pschung@dankook.ac.kr (P.S.C.); 3 Beckman Laser Institute Korea, Dankook University College of Medicine, Cheonan 31116, Korea; 4 Laser Translational Clinical Trial Center, Dankook University Hospital, Cheonan 31116, Korea

**Keywords:** allergic rhinitis, low-level laser therapy, randomized controlled trial, quality of life, treatment

## Abstract

Allergic rhinitis (AR) is a common disease that interferes with the daily activities and reduces the quality of life. Conventional treatments often do not provide complete resolution of the symptoms, and many new treatment modalities have been tried. This study aimed to evaluate the efficacy and safety of low-level laser therapy (LLLT) for AR in a randomized, double-blind, placebo-controlled trial. Patients diagnosed with AR were randomly allocated to receive LLLT or sham treatment. The primary outcome was a change in the reflective total nasal symptom score (TNSS). The secondary outcome was quality of life scores assessed using the Rhinoconjunctivitis Quality of Life Questionnaire. Incidences of adverse events were also recorded. Among 67 randomized subjects, 41 subjects (22 in LLLT group and 19 in sham treatment group) were included for efficacy analysis. The LLLT group showed a significantly improved TNSS score compared to the sham treatment group for decreasing AR symptom severity (*p* = 0.011) and improving quality of life regarding nasal symptoms (*p* = 0.036) at the end of treatment. Throughout the treatment period, no severe adverse events occurred. This clinical trial showed that LLLT is an effective and safe option for the management of AR regarding symptom relief and quality of life improvement.

## 1. Introduction

Allergic rhinitis (AR) is a chronic airway disorder and one of the most common health problems [1]. AR is associated with a substantial health and psychological burden to patients due to its high incidence, etiologically complex, and prolonged disease course [2,3]. AR is characterized by nasal symptoms such as congestion, sneezing, rhinorrhea, and itching [4]. Although these symptoms are usually considered nonserious, they do affect the daily and social activities of an individual; they sometimes have significant effects on the quality of life and even result in considerable medical expenses [5,6]. In addition, the prevalence of perennial AR has been increasing in recent years [7].

The standard treatment for AR includes patient education, avoidance of allergen, medical treatment, and immunotherapy. However, these treatments frequently do not provide complete resolution of the symptoms. Adverse effects of the medications include drowsiness and dryness of the throat, which can disturb the patient’s quality of life almost as much as the symptoms they are trying to alleviate [8].

Among several trials regarding the treatment of AR, low-level laser therapy (LLLT) has been reported to reduce the symptoms of AR and improve the quality of life [9,10,11,12,13,14,15]. Furthermore, evidence of its anti-inflammatory and immunosuppressive effects has also been reported [16,17]. LLLT does not only improve AR symptoms, but also decreases nasal mucosal interleukin (IL)-5 levels and reduces the eosinophil count [10]. In an animal study, a low-level laser (658 nm, 30 mW) was irradiated into the intranasal cavity of an ovalbumin-induced AR mouse model. It proved effective in inhibiting total immunoglobulin E and IL-4, and it was confirmed by histological studies to have reduced the number of infiltrated inflammatory cells in the intranasal epithelium [18]. However, previous studies had limitations in studying only moderate to severe AR patients, and allowing rescue medicine that could make interpretation of the results difficult. Therefore, there was a need for a study conducted with a larger sample under a more rigorous protocol.

In this study, we tried to clarify the efficacy and safety of LLLT in a randomized double-blind placebo-controlled trial among Korean patients with perennial AR.

## 2. Materials and Methods

### 2.1. Setting

A randomized, double-blind, and placebo-controlled study was designed to evaluate the clinical efficacy and safety of LLLT for AR treatment. The study was conducted at the Otorhinolaryngology Department of the Dankook University Hospital, Korea. The trial period was from April 2017 through March 2018. The study protocol was approved by the Institutional Review Board of Dankook University Hospital (IRB no. 2016-09-011) and was registered in the clinical research information system of South Korea (CRIS Registration Number KCT0003291). The study was conducted in accordance with the principles of the Declaration of Helsinki.

### 2.2. Sample Size Calculation

We established a sample size for an independent *t*-test using the adjusted standard deviation and the difference in total nasal symptom score (TNSS) change between the LLLT and the sham-controlled group. The effect size estimate was calculated using the results of our previous pilot study with a sample size of 40 (not published). The TNSSs in the LLLT and sham-controlled group were 2.462 and 0.786, respectively, and the difference in TNSS change between two groups was 1.676. The standard deviation of the pilot study was adjusted for better application to the true population. With participants recruited in a 1:1 ratio in the two groups, the sample size required to yield 80% power with a two-tailed significance level of 5% was calculated as 31 participants per group. To evaluate the treatment effect at the end of treatment and consider dropouts, the potential recruits were increased to up to 34 participants in each group.

### 2.3. Participants

Participants suffering from perennial AR were recruited. The inclusion criteria were: (1) participants aged older than 19 years; (2) participants suffering from mild to moderate symptoms at week 0 (baseline TNSS ≤ 9) of AR (nasal congestion, rhinorrhea, sneezing, and nasal itching) and a proven positive allergy test (skin prick test or multiple allergen simultaneous test) to any of the common perennial allergens such as dust mites, molds, insects, and animal danders within the last 12 months; (3) participants with any gender or ethnicity; and (4) participants who signed written informed consent before they were assigned. The exclusion criteria were as follows: (1) patients younger than 18 years of age; (2) pregnant or breastfeeding women; (3) patients with definite deviated nasal septum, or sinusitis or history of operation within the last 6 months; (4) patients with hypertension, diabetes mellitus, malignancy, anemia, active pulmonary tuberculosis, infection, active respiratory disease like asthma, or other systemic diseases; (5) patients with long-term use of corticosteroids or immunosuppressive agents; (6) patients with hypersensitivity to laser or a wound at the laser site; (7) patients who were involved in another clinical study within 30 days; (8) patients who were unable to comply with the follow-up schedules; and (9) patients who had used antihistamines within 1 week, topical corticosteroids within 2 weeks, systemic corticosteroids within 4 weeks, anti-cholinergic drugs within 3 days, anti-leukotriene drugs within 1 week, decongestants within 3 days, tricyclic antidepressants or phenothiazines within 2 weeks, non-steroidal analgesics within 2 weeks, and other drugs which the researchers believed were inappropriate.

### 2.4. Randomization and Blinding

Random numbers were generated by an independent statistician using a computer-based random assignment procedure. Using these random numbers, a LLLT device or sham treatment device was distributed to each participant. The sham treatment device was visually identical to the LLLT device, so neither the participants nor the physicians were aware of the list of participants in the LLLT and sham treatment groups. All participants were informed that there was a 50% chance of receiving the LLLT or sham treatment. In addition, trial data were analyzed by a statistician who was blinded to the participants’ allocation.

### 2.5. Intervention

**Figure 1:**
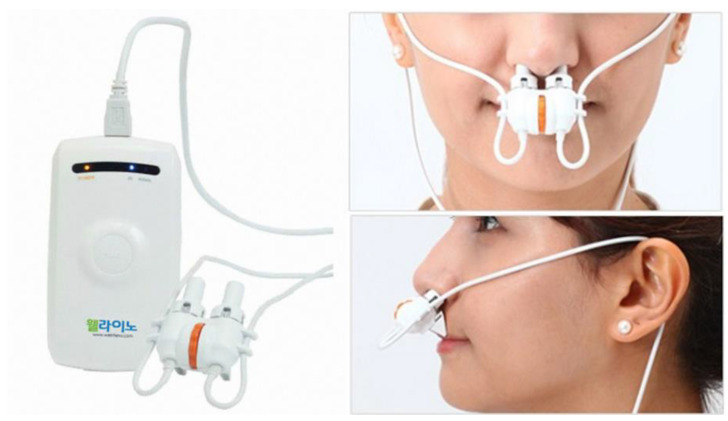
Device for the intranasal laser irradiation (Optowell^®^, Jeonju, Korea). The device was applied intranasally for laser irradiation.

After screening, participants who had taken the medication had washout periods and the duration of washout periods was up to 1 month, which was determined by the type of the medication. During washout period, the participants stopped taking medication. At the end of the run-in period, participants were randomized into either the LLLT group or sham treatment group by a computer-generated random number allocation scheme. The participants were treated using LLLT (multi-wavelength; AlGaInP 670 nm, 3 mW, GaAs 830 nm, 20 mW; Optowell Co. Ltd., Jeonju, Korea) or sham treatment for 20 min twice a day (morning and evening) for 4 weeks (Figure 1, Table 1). The parameters used for laser irradiation in the present study are shown in Table 2. The LLLT and sham treatment device was identical in shape with different parameters and has a memory that can check the usage time. Compliance was assessed based on device usage log data and usage records in the distributed diary of each participant. In this study, the participants were required to cease any medication for AR and were instructed not to use any relief medication. The intervention was terminated in cases of severe adverse events, voluntary withdrawal of participants from the study, and unpermitted medication use.

### 2.6. Primary Outcome

The primary outcome was the change in TNSS [19] from baseline to the end of treatment. The primary efficacy indicators included four common symptoms of AR: nasal congestion, rhinorrhea, sneezing, and nasal itching. Each symptom was scored from 0 to 4 according to the severity (0, no symptoms; 1, mild symptoms, such as symptoms that were present but not particularly bothersome; 2, moderate symptoms, such as symptoms that were bothersome but did not interfere with daily activities; 3, severe symptoms such as symptoms that were bothersome and interfered with daily activities or disturbed sleep; 4, very severe symptoms) over the previous 12 h time interval. The TNSS was calculated by summing the score for each of the symptoms. This questionnaire was completed by the participants at week 0 (baseline) and 4.

### 2.7. Secondary Outcome

The secondary outcome indicators included the quality of life scored according to the Rhinoconjunctivitis Quality of Life Questionnaire (RQLQ) [20]. The RQLQ is a self-reported questionnaire which is categorized into seven areas with 28 questions regarding daily activities, sleeping state, practical problems, nasal symptoms, eye symptoms, non-nasal/eye symptoms, and emotional function domains. The participants were asked to recall their experiences during the preceding week and to rate each answer using a 7-point scale (0, no impairment; 6, severe impairment). This questionnaire was completed by participants at week 0 (baseline) and week 4.

### 2.8. Adverse Events

At each visit, the participants reported adverse events, including the specific symptom, onset, severity, duration, time of resolution, and possible association with treatment. Possible adverse events were nasal dryness, epistaxis, burning, inflammation, and nasal septum perforation. All adverse effects were analyzed at the study endpoint, regardless of whether they were considered relevant to the treatment or not. Details of the participants who withdrew due to serious adverse events were reported according to the reporting requirements.

### 2.9. Statistical Analysis

The statistical analysis was performed by a statistician blinded to the group allocation. Data were summarized as frequencies for categorical variables and means and standard deviations for continuous variables. Baseline demographic characteristics, such as sex and family history, were analyzed using the chi-squared test or an independent-samples *t*-test as appropriate.

Per-protocol population was defined as a subset of an intention-to-treat (ITT) population by excluding two subjects who took unpermitted medications during the trial. In addition, subjects whose medical device compliance was <50% were excluded from the per-protocol set, and this population was defined as the modified per-protocol set. Efficacy analysis was performed using the modified per-protocol (mPP) set, and safety was analyzed using the per-protocol set.

For primary outcome measures, analysis of covariance with the value before administration (ANCOVA) was performed using modified per-protocol set, and the least square means (LSMeans) of changes in each TNSS variation from baseline, adjusted for bias before treatment, were determined for each treatment group. Then, intergroup differences in LSMeans and 95% CIs during the observation period were calculated. For the primary end point, a pairwise test (between-group comparison) was performed using the above ANCOVA model and the PROC MIXED procedure in Statistical Analysis System (SAS; version 9.2, SAS Institute, Cary, NC, USA) to verify the superiority of LLLT over sham treatment. Values of *p* < 0.05 were considered statistically significant. For secondary outcome measures, the two-samples independent *t*-test or the Wilcoxon rank-sum test was used to compare continuous variables as appropriate, and the chi-squared test was used to compare categorical variables between the two groups.

## 3. Results

### 3.1. Participants and Baseline Characteristics

**Figure 2:**
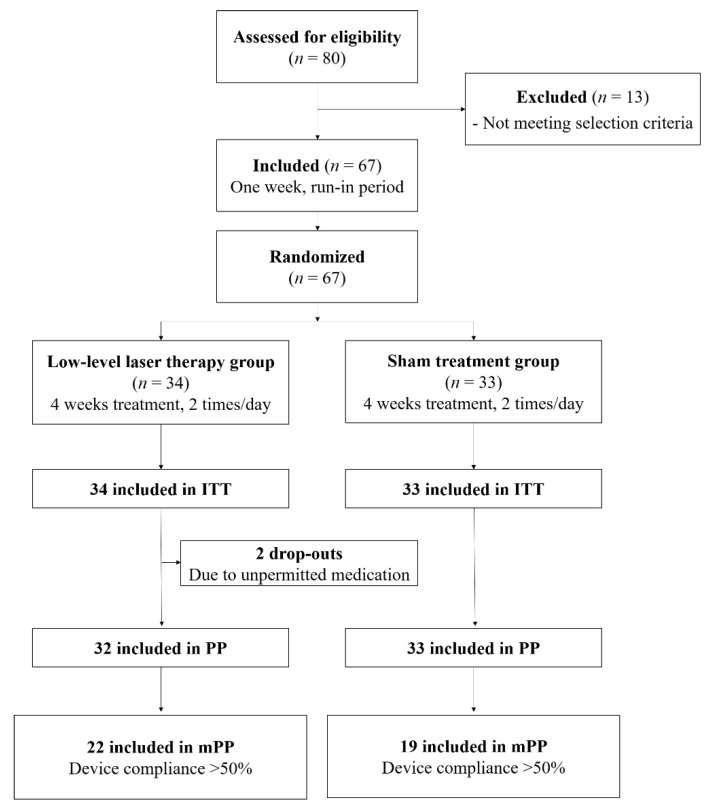
Flow diagram of clinical trial and disposition of patients. Abbreviations: ITT, intent to treat set; PP, per-protocol set; mPP, modified per-protocol set.

Eighty participants were screened via enquiry. Thirteen participants who did not meet the inclusion criteria were excluded; therefore, 67 participants (ITT set) were included in the trial and were randomized to the LLLT group (*n* = 34) or sham treatment group (*n* = 33). No significant differences were observed between the groups regarding sex, underlying health status, and laboratory test results. However, there was a significant difference in the mean age between the two groups (27.82 ± 6.74 years for the LLLT group and 33.79 ± 12.28 years for the sham treatment group, *p* = 0.018). There were no significant differences in TNSS or RQLQ between the two groups at baseline (*p* > 0.05, Table 3). During the four week treatment period, two participants in the LLLT group discontinued due to unpermitted medication use; therefore, of the 67 participants in the ITT group, 65 participants (per-protocol (PP)) were defined. Among the 65 patients in the PP set, 10 in the LLLT group and 14 in the sham treatment group had less than 50% compliance. Excluding these participants with lower compliance, a total of 41 participants (22 in the LLLT group and 19 in the sham treatment group) were categorized as the modified per-protocol (mPP) set (Figure 2).

### 3.2. Primary Outcome

**Figure 3:**
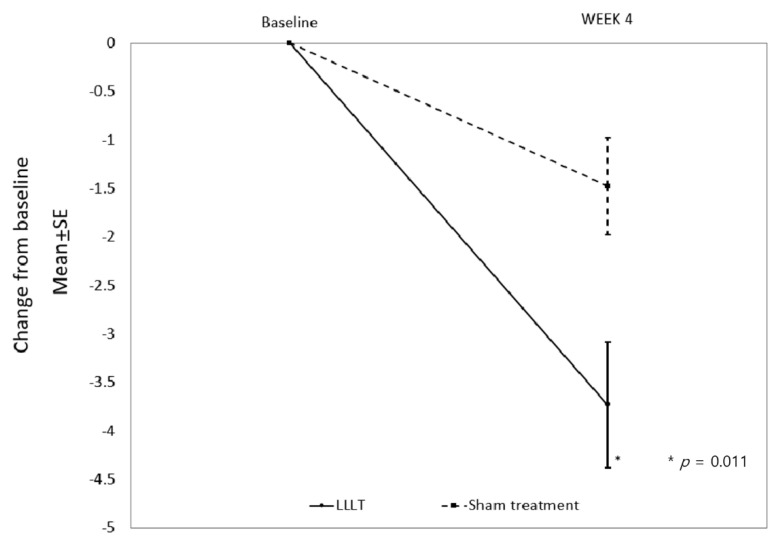
Least square means (LSMeans) in the TNSS from baseline to the end of treatment.

mPP analysis showed that both the LLLT and sham treatment groups had significantly reduced TNSSs at the end of the treatment period compared with the baseline (7.05 ± 3.11 to 3.32 ± 2.85 in the LLLT group, *p* < 0.001; and 6.21 ± 2.15 to 4.74 ± 2.35 in the sham treatment group, *p* = 0.009, Table 4). There was a greater reduction in TNSS in the LLLT group than in the sham treatment group (*p* = 0.011, Table 4). The primary end point was a change (LSMeans ± SE) in the TNSS from baseline to the end of treatment, the values for which were −3.72 ± 0.64 and −1.47 ± 0.50 in the LLLT and sham treatment groups, respectively (Figure 3). The between-group difference ((LLLT group)–(sham treatment group)) was −2.25 (95% CI: −3.948 to −0.559, *p* = 0.011), which validated the hypothesis that LLLT was superior to a placebo in terms of efficacy.

### 3.3. Secondary Outcome

In the LLLT group, the RQLQ score was 60.50 ± 27.43 at the baseline and decreased to 39.05 ± 27.01 at the end of the treatment period (*p* < 0.001). In the sham treatment group, the RQLQ score was 57.63 ± 25.07 at baseline and decreased to 41.37 ± 26.92 (*p* < 0.001). A greater reduction in RQLQ score was noted in the LLLT group without statistical significance (*p* = 0.383, Table 5).

**Figure 4:**
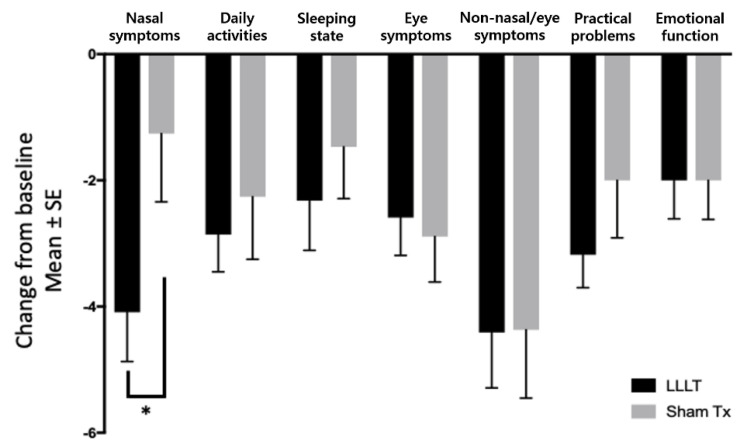
Seven domains of the RQLQ change from baseline. * *p* < 0.05.

Among the seven domains of the RQLQ, there were no significant differences in the reduction in following domains between groups: daily activities (difference = 0.60, *p* = 0.596), sleeping state (difference = 0.84, *p* = 0.464), eye symptoms (difference = 0.30, *p* = 0.745), non-nasal/eye symptoms (difference = 0.04, *p* = 0.977), practical problems (difference = 1.18, *p* = 0.272), and emotional function (difference = 0.001, *p* = 1.000). However, in the nasal symptoms domain, the mPP analysis showed that the RQLQ score was significantly reduced from 11.27 to 7.18 (difference = 4.09) in the LLLT group, and 9.63 to 8.37 (difference = 1.26) in the sham treatment group (*p* = 0.036; 95% CI, −5.464, −0.191, Table 6), suggesting that LLLT improves nasal symptoms (Figure 4, Table 6).

### 3.4. Adverse Events

Safety analysis was performed in the PP set. Some adverse events were reported by the participants during the treatment period (three in the LLLT group, five in the sham treatment group). In the LLLT group, all of the events were classified as definitely unrelated to the treatment. In the sham treatment group, 60.0% (*n* = 3) of the events were classified as definitely unrelated to the treatment, and 40% (*n* = 2) as possibly unrelated (Table 7). There was no significant difference in the frequency of adverse events between the two groups (*p* = 0.476). No serious adverse events were reported or observed during the study.

## 4. Discussion

This randomized, double-blind, placebo-controlled trial revealed that LLLT significantly suppressed the clinical symptoms of perennial AR at the end of the four-week treatment. The symptoms were improved to a greater extent in the LLLT treatment group. In addition, participants who received LLLT reported their quality of life regarding nasal symptoms to be much improved compared to those who received the sham treatment. In addition, there was no difference in the number of participants who reported adverse events, and there were no dropouts due to adverse events. Based on these findings, we recommend LLLT for the control of AR symptoms.

The mechanisms underlying LLLT have not been fully understood. However, possible mechanisms of action of LLLT in the treatment of AR have been studied previously [10,13,14,15,16,17,18]. The benefits of LLLT in AR may be primarily explained by anti-inflammatory mechanisms [21,22]. In animal models of acute pulmonary inflammation, LLLT relieved airway inflammation through the induction of IL−10 and reduction in the expression of macrophage inflammatory protein 2 and tumor necrosis factor (TNF) [23]. Koreck et al. reported that irradiation of the nasal mucosa resulted in a local reduction in IL-5, a cytokine that promotes the activation, maturation and prolonged survival of eosinophils, which are the main effector cells in AR [10]. Low-level lasers have been irradiated into the intranasal cavity of an ovalbumin-induced AR mouse model. Its inhibitory effect on immunoglobulin E and IL-4 production was histologically demonstrated via a reduced number of infiltrated inflammatory cells in the intranasal epithelium [18]. A review article concluded that LLLT reduces the concentration of signal molecules involved in the inflammatory response, and that LLLT can inhibit TNF-α, cyclooxygenase-2, prostaglandin E2, and IL-1β [24]. All these effects of LLLT are desirable for improving symptoms among patients with AR.

Clinically, Csoma et al. showed that 308 nm of ultraviolet B irradiation significantly minimized AR symptoms, including nasal congestion, rhinorrhea, and sneezing, and improved the total nasal symptom scores and allergen-induced skin prick test results [25]. In a previous study, Garaczi et al. even insisted that intranasal irradiation with a combination of low-dose ultraviolet B and visible light was significantly better than fexofenadine hydrochloride for the treatment of seasonal AR [26]. In a pilot study conducted in Korea that included 42 patients with perennial AR, nasal LLLT with a 650 nm laser significantly reduced the symptoms of AR [12]. In previous studies, the participants were patients with moderate to severe AR. However, the participants of this study had mild to moderate AR with a TNSS ≤9. In addition, in contrast to the aforementioned studies, the participants in this study stopped any medication prior to enrollment in the study and were not allowed to use any AR symptom relievers throughout the course of treatment.

Dryness of the nasal mucosa, erythema, skin pain, pruritus, and pigmentation are potential LLLT treatment-specific adverse events mentioned in previous studies [10,13,15]. Dryness was not severe, did not increase the likelihood of nasal bleeding, and was easily controlled with emollients 2–3 times a day. Furthermore, it has been reported that dryness does not last after the end of treatment. In this study, none of the patients experienced dryness. Additionally, thermal effects may be a concern; however, low-level laser is a form of light emission with a power output of less than 500 mW, and is considered as a type of nonthermal irradiation to living tissue. Another concern was that ultraviolet light, which can be mutagenic and carcinogenic, may cause DNA damage [27,28]. However, several studies have shown that nasal mucosae exposed to ultraviolet light have the capacity to repair DNA damage, which suggests that the multistep process of carcinogenesis has not been triggered, and no residual damage was seen in human subjects exposed to multiple ultraviolet B treatments [15,29,30]. Mitchell et al. showed that the nasal mucosa was capable of efficient repair of ultraviolet-induced DNA damage and suggested that ultraviolet phototherapy can be used in the treatment of AR [29]. The visible and infrared light used in this study have been shown to be safe. In addition, lasers with wavelengths of 700–1400 nm are classified in the “retinal hazard region”; however, in this study, we only used 3 mW and 20 mW lasers, which are not hazardous to the eye.

This study had some limitations. Poor compliance in the use of the device for the trial (<50%) was observed in both groups (LLLT group, *n* = 10 (31.3%); and sham treatment group, *n* = 14 (42.4%)). In the clinical trial of the newly developed device, it was important to evaluate the effect when compliant to some extent. Therefore, participants with poor compliance were judged as having non-sufficient exposure to study treatment and excluded, and it was thought that this was the basis for the use of mPP analysis to evaluate efficacy in more optimal conditions. In addition, there was no difference in compliance between the two groups of mPP. In the final mPP analysis, the sample size estimated was not achieved, and the verification power was statistically lowered. The reasons for the poor compliance could be speculated as follows; firstly, the AR symptoms among the participants were mild to moderate, which might have contributed to the poor compliance. Secondly, the 20 min usage time twice per day with the device in the nose could bother the participants to make them use it less. With the efforts to improve the compliance of device, more verifiable study and statistical analysis should be conducted later. The second limitation was the short follow-up period. The structural changes and biochemical activity induced by the procedure would reduce inflammation in the tissue but resolve later. Therefore, additional studies with longer follow-up periods are needed to evaluate the duration of the effects of LLLT. In addition, although LLLT appears to be safe and well tolerated, it was unclear if long-term low-level laser irradiation or thermal exposure of the nasal mucosa could result in DNA damage or irreversible changes. Long-term follow-up studies should be conducted in the future. Thirdly, treatment was only administered for four weeks, and the optimal duration for treatment with LLLT is unknown. Thus, it is possible that shorter durations could have a similar effect, and that longer use of the treatment or repeated treatments may enhance the outcome. Further studies are required to verify this. The fourth limitation was TNSS changes from the baseline at the final assessment was used in this study. The FDA recommended the change from baseline in the TNSS averaged over the entire treatment period as the primary endpoint. However, several other clinical trials also used TNSS changes, like in this study [31,32]. Despite these limitations, the present study was conducted according to a rigorous protocol, with a relatively large sample in a double-blind randomized and sham-controlled trial. In this regard, the results of this study are meaningful and encouraging.

## 5. Conclusions

We have demonstrated a significant reduction in the symptoms of perennial AR and an improved quality of life regarding nasal symptoms in patients treated with LLLT compared to those treated with the sham treatment. The results suggest that LLLT might be an effective and safe treatment for controlling the symptoms of AR.

## Figures and Tables

**Table 1:** Laser specifications used in low-level laser therapy.

Laser Wavelength	Output Power	Gain Medium
670 nm (visible ray)	670 nm (visible ray): 3 mW (1 mW, 3 each)	670 nm: AlGaInP
830 nm (infrared ray)	830 nm (infrared ray): 20 mW	830 nm: GaAs

**Table 2:** Parameters of the irradiation device.

Product	Wellrhino
Light source	VCSEL
Wavelength	670 and 830 nm
Laser power	670 nm, 3 mW; 830 nm, 20 mW
Total power	23 mW
Using Time	20 and 40 min

**Table 3:** Comparison of participant characteristics in the two groups (intention-to-treat).

Variable	LLLT (*n* = 34)	Sham Treatment (*n* = 33)	*p*-Value
Age (years)	27.8 ± 6.7	33.8 ± 12.3	0.018
Gender (male:female)	17:17	13:20	0.383
TNSS	5.94 ± 3.29	6.64 ± 2.06	0.243
RQLQ	58.29 ± 26.06	61.39 ± 22.91	0.433
Daily activities	7.00 ± 2.76	7.97 ± 3.12	0.066
Sleeping state	6.53 ± 4.42	6.00 ± 3.30	0.935
Eye symptoms	7.18 ± 4.98	7.55 ± 5.16	0.748
Non-nasal/eye symptoms	13.65 ± 7.55	13.79 ± 6.27	0.665
Practical problems	7.76 ± 3.64	8.73 ± 3.77	0.304
Emotional function	5.53 ± 4.30	6.55 ± 3.76	0.174
Nasal symptoms	10.65 ± 4.66	10.82 ± 3.90	0.823

Abbreviations: LLLT, low-level laser therapy; TNSS, total nasal symptom score; RQLQ, Rhinoconjunctivitis Quality of Life Questionnaire. Values are expressed as mean ± standard deviation. The *p*-values were obtained from a chi-squared test and Wilcoxon’s rank-sum test.

**Table 4:** Treatment effects (TNSS, mPP set).

Group	Baseline	End of Treatment	Difference	Difference between Groups: *p*-Value
LLLT (*n* = 22)	7.05 ± 3.11	3.32 ± 2.85	−3.73 ± 3.03	0.011
Sham treatment (*n* = 19)	6.21 ± 2.15	4.74 ± 2.35	−1.47 ± 2.20	

Abbreviations: TNSS, total nasal symptom score; LLLT, low-level laser therapy; mPP set, modified per-protocol set. Values are expressed as mean ± standard deviation. The *p*-values were obtained from a chi-squared test and Wilcoxon’s rank-sum test.

**Table 5:** Treatment effects (RQLQ, mPP set).

Compliance	Group	Baseline	End of Treatment	Difference	Comparison of Difference between Groups: *p*-Value
(Modified Per-Protocol)	LLLT (*n* = 22)	60.50 ± 27.43	39.05 ± 27.01	−21.45 ± 14.79	0.383
	Sham treatment (*n* = 19)	57.63 ± 25.07	41.37 ± 26.92	−16.26 ± 22.58	

**Table 6:** Treatment effects (RQLQ domains, mPP set).

Domains	Group	Baseline	End of Treatment	Difference	*p*-Value
Nasal symptoms	LLLT (*n* = 22)	11.27 ± 4.41	7.18 ± 4.24	−4.09 ± 3.65	0.036
	Sham treatment (*n* = 19)	9.63 ± 3.35	8.37 ± 4.65	−1.26 ± 4.69	
Daily activities	LLLT (*n* = 22)	7.32 ± 2.71	4.45 ± 2.94	−2.86 ± 2.78	0.596
	Sham treatment (*n* = 19)	7.74 ± 3.36	5.47 ± 3.88	−2.26 ± 4.33	
Sleeping state	LLLT (*n* = 22)	7.09 ± 4.47	4.77 ± 3.78	−2.32 ± 3.68	0.464
	Sham treatment (*n* = 19)	5.84 ± 3.73	4.37 ± 3.68	−1.47 ± 3.60	
Eye symptoms	LLLT (*n* = 22)	6.95 ± 5.72	4.36 ± 4.48	−2.59 ± 2.81	0.745
	Sham treatment (*n* = 19)	6.89 ± 5.30	4.00 ± 3.57	−2.89 ± 3.14	
Non-nasal/eye symptoms	LLLT (*n* = 22)	14.18 ± 7.76	9.77 ± 7.37	−4.41 ± 4.15	0.977
	Sham treatment (*n* = 19)	13.32 ± 6.38	8.95 ± 6.39	−4.37 ± 4.73	
Practical problems	LLLT (*n* = 22)	7.82 ± 3.25	4.64 ± 3.65	−3.18 ± 2.46	0.272
	Sham treatment (*n* = 19)	8.05 ± 3.26	6.05 ± 4.14	−2.00 ± 3.99	
Emotional function	LLLT (*n* = 22)	5.86 ± 4.55	3.86 ± 3.72	−2.00 ± 2.88	1.000
	Sham treatment (*n* = 19)	6.16 ± 4.30	4.16 ± 4.11	−2.00 ± 2.71	

Abbreviations: RQLQ, Rhinoconjunctivitis Quality of Life Questionnaire; LLLT, low-level laser therapy; mPP set, modified Per-Protocol set. The *p*-values were obtained from a chi-squared test and Wilcoxon’s rank-sum test.

**Table 7:** Reported adverse events associated with LLLT and sham treatment participants.

Group	Adverse Events (*n*)
LLLT group	Definitely unrelated: idiopathic sudden hearing loss (1), injury of unrelated body part (1), pain of unrelated body part (1)
Sham treatment group	Definitely unrelated: dyspepsia (1), pain of unrelated body part (1), incidental finding of unrelated body part (1) Possibly unrelated: acne (1), upper respiratory infection (1)

## Data Availability

Not applicable.
